# Effects of Microsatellite Instability on the Clinical and Pathological Characteristics of Colon Cancer and the Diagnostic Accuracy of Preoperative Abdominal CT Scans

**DOI:** 10.3390/diagnostics15020190

**Published:** 2025-01-15

**Authors:** Rıdvan Yavuz, Orhan Aras, Hüseyin Çiyiltepe, Onur İlkay Dinçer, Ahmet Şükrü Alparslan, Tebessüm Çakır

**Affiliations:** 1 Gastroenterology Surgery Department, Antalya Training and Research Hospital, Varlık, Kazım Karabekir Cd., Muratpaşa 07100, Antalya, Turkey; drorhanaras@hotmail.com (O.A.); drciyiltepe@hotmail.com (H.Ç.); onurdncr@hotmail.com (O.İ.D.); cakir.tebessum@gmail.com (T.Ç.); 2 Radiology Department, Antalya Training and Research Hospital, Varlık, Kazım Karabekir Cd., Muratpaşa 07100, Antalya, Turkey; ahmet_alparslan@yahoo.com

**Keywords:** colon cancer, microsatellite instability, computed tomography, preoperative staging, systemic inflammatory markers, C-reactive protein

## Abstract

**Background**: Microsatellite-stable (MSS) and microsatellite-instable (MSI) colon cancer (CC) cases have different characteristics. These characteristics may impact the accuracy of abdominal computed tomography (CT) scan examinations in MSI CC. **Methods**: A retrospective analysis was conducted to examine the effects of MSI CC on patients’ clinical and tumor characteristics. We determined the accuracy of radiological T and N staging compared to pathological T and N staging in CC patients and evaluated the influence of tumor- and patient-related factors on this accuracy. **Results**: A total of 131 CC patients who had undergone surgical resection were analyzed. Mismatch repair-deficient (dMMR) CC was predominantly found in the right hemicolon (*p* = 0.023); it was more likely to exhibit moderate (80.8%) or low-grade differentiation (*p* = 0.01) and had higher rates of mucinous differentiation (*p* = 0.001). The median neutrophil and platelet counts and C-reactive protein (CRP) levels at diagnosis were significantly higher in patients with dMMR CC (*p* = 0.022, *p* = 0.022, and *p* = 0.018). The depth of invasion influenced the CRP levels in dMMR CC cases (*p* = 0.015). The abdominal CT exam was accurate regarding the depth of colonic wall invasion in 58.1% and 38.5% of patients with mismatch repair-proficient (pMMR) and dMMR CC, respectively. The assessment of lymph node invasion was accurate in 44.8% of those with pMMR and 50.0% of those with dMMR CC. There was no significant difference in the accuracy in predicting the T and N statuses between the two groups. The accuracy in the determination of the T and N statuses was not affected by the parameters examined. **Conclusions**: dMMR CC has specific characteristic features. MSI does not affect the accuracy of preoperative abdominal CT.

## 1. Introduction

Colon cancer (CC) is the second-leading cause of cancer-related deaths worldwide [1]. According to the WHO data, by 2040, the burden of CC will increase to 3.2 million new cases per year (an increase of 63%) and 1.6 million deaths per year (an increase of 73%) [1]. Although widespread screening programs exist, only 20–25% of patients are diagnosed in the asymptomatic phase [2,3]. Consequently, most patients diagnosed with this cancer require advanced treatment [4]. Microsatellite instability (MSI) results in distinct genotypic and phenotypic subgroups, which account for 12–15% of all colorectal cancers [5]. In the 1990s, distinct genetic alterations reflecting the amplification or deletion of interspersed dinucleotide, trinucleotide, and other simple repeat sequences were detected in colorectal cancer. Elements of this type were referred to as microsatellites, and these constitute one of the most abundant classes of repetitive DNA families in the human genome [6]. Microsatellite instability may occur sporadically or be inherited in an autosomal dominant fashion [7]. Colon cancers with MSI exhibit distinct features in terms of the proximal location [8,9], differentiation [10,11], and mucinous histopathology [12]. Additionally, MSI leads to an increase in intraepithelial lymphocytes and pronounced lymphocytic infiltration within the tumor, resembling Crohn’s disease [13,14].

Neoadjuvant treatments are well accepted and are generally performed in cases of gastric, esophageal, and rectal cancers. Despite accumulating evidence regarding the benefits of preoperative chemotherapy in colon cancer, neoadjuvant chemotherapy (NAC) is recommended only as an alternative treatment for clinical T4 patients in the NCCN [15] and Korean guidelines [16], and it is weakly recommended for resectable metastatic disease in the ESMO guidelines [17]. On the other hand, a recent meta-analysis involving 2120 CC patients demonstrated that NAC is associated with a reduced risk of recurrence and death, as well as improved overall survival [18]. Seven studies were included in this meta-analysis; however, only two of them analyzed the MMR [19,20] status. A recent phase III trial evaluating the preoperative FOxTROT treatment in operable CC, radiologically staged as T3–4 and N0–2, demonstrated similar findings [20]. In this study, moderate or greater regression after NAC was seen in only 7% of dMMR CC cases, compared with 23% of pMMR CC cases. One of the main concerns regarding NAC is that the limited accuracy of the preoperative radiological evaluation can lead to overtreatment [21], unnecessary exposure to chemotherapy-related toxicity, and an increased risk of perioperative complications [22]. Various studies have compared preoperative abdominal CT scan examinations with postoperative pathological reports [23]. One study, which examined a total of 59,558 patients from the Netherlands’ national colorectal cancer database, reported an average accuracy of 75.4% for T staging and 69.5% for N staging [24]. Another national database study involving 3749 patients classified as having pathological T3–4 tumors found that 27.0% of these cases were understaged. Among 3044 patients who represented true lymph node-negative cases (pN0), 33.8% were overstaged via CT scans [25]. Elucidating the factors that contribute to these limitations is critical in order to improve patient-specific decision making regarding neoadjuvant treatment.

MSI is expected to limit the accuracy of preoperative evaluations due to the profound immune reaction around the tumor and lymph nodes [12,26]. Limited studies have evaluated the effect of the MSI status on radiological evaluations. A few studies comparing preoperative CT scan results with pathological evaluations have shown increased overstaging in lymph node assessment [27,28]. Platt et al. also demonstrated a relationship between increased systemic inflammation markers and the presence of lymph node metastases, highlighting the greater risk of N overstaging in dMMR patients [29]. dMMR groups exhibit distinct CT scan characteristics, such as increased tumor and lymph node diameters [11,30], greater hypoenhancement, and increased inhomogeneity [11].

Our primary aim was to describe the patient- and tumor-related differences in microsatellite-stable and -instable CC. The secondary aim was to assess the concordance of preoperative CT scan examinations with postoperative pathological evaluations and elucidate the patient- and tumor-related factors that affect this concordance, including the MMR status.

## 2. Materials and Methods

### 2.1. Study Design

This was a retrospective analysis designed to examine the effects of MSI on tumor characteristics, determine the accuracy of radiological T and N staging compared to pathological T and N staging in CC patients, and evaluate the influence of tumor- and patient-related factors on this accuracy. The studied patient-related factors included gender, age, body mass index (BMI), baseline peripheral blood neutrophil count, platelet count, and C-reactive protein (CRP). The tumor-related factors were the tumor side, differentiation grade, presence of a mucinous component, depth of colonic wall invasion, lymph node invasion, and MMR status. The cases were not restaged during the study period. As this was a retrospective evaluation of routine and anonymized patient data, written consent was not required. Approval for the project was obtained from the Ethics Committee of the University of Health Sciences, Antalya Training and Research Hospital (2024-037).

### 2.2. Patient Selection

All patients aged between 30 and 85 years who had undergone curative surgical resection for colon cancer between January 2021 and 1 April 2024 in a specialized gastroenterological surgery unit were analyzed. Patients were eligible for inclusion if they had a histological diagnosis of CC and documented radiological staging, postoperative pathological staging (according to TNM version 8; T1 to T4, N0 to N2, and M0), and MMR status data (obtained via four-protein immunohistochemistry). Patients were excluded if they had metastatic disease in the preoperative radiological or postoperative pathological examination, an unknown MMR status, or tumors localized in the rectosigmoid or rectum. Patients evaluated with preoperative non-contrast CT scans or MRI examinations were also excluded.

### 2.3. Data Collection

**Figure 1:**
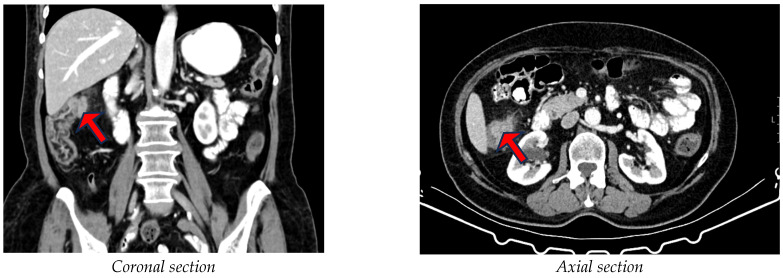
Abdominal CT scan showing colonic wall infiltration. Arrows show T3 tumor in coronal and t3 tumor in axial section.

**Figure 2:**
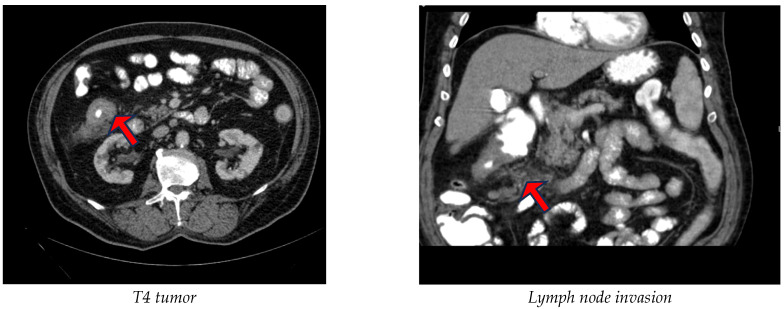
Abdominal CT scan showing T4 tumor and lymph node invasion. Arrows show T4 tumor and Lymph node invasion.

We collected data on age, gender, BMI, baseline peripheral blood neutrophil counts, platelet counts, and CRP levels from the hospital database. The tumor location was extracted from the operative notes. Abdominal CT scans were evaluated based on radiology reports. In our routine practice, a thoracoabdominal CT is the standard imaging modality for the preoperative staging of colon cancer patients. All CC patients’ abdominal and thoracic CT scans were preoperatively reviewed by the same gastrointestinal radiologist and evaluated regarding T and N staging to determine the necessity of neoadjuvant treatment (Figure 1 and Figure 2). The histological subtype (adenocarcinoma or mucinous adenocarcinoma), tumor grade (poorly, moderately, or well differentiated), and pathological stage (T, N, and M where appropriate) were collected from pathology reports. An upfront analysis of the MMR status using immunohistochemistry (IHC) for MLH1, PMS2, MSH2, and MSH6, according to standard protocols, has been routinely performed for all colorectal tumors in our hospital since January 2021. The pMMR status was characterized by considering the full expression of all four proteins, while dMMR was characterized by the lack of expression of at least one of these proteins.

### 2.4. Statistics

The patients’ baseline characteristics were explored to identify significant differences between the dMMR and pMMR groups. Data from all patients were recorded in an Excel spreadsheet. Statistical analysis was performed using the Statistical Package for the Social Sciences (SPSS, Chicago, IL, USA), version 24.0. Continuous variables were presented as the median (interquartile range, IQR) and mean (standard deviation, SD), and categorical variables were presented as numbers (percentage, %). The normality of the data was assessed using the Shapiro–Wilk test. Student’s *t*-test was used to compare parametric variables. The Mann–Whitney U test was used to compare non-parametric continuous variables. Categorical data were assessed using the Pearson chi-squared test. *p*-values < 0.05 were considered statistically significant. The radiological T and N stages were considered correct if they matched the pathological staging. Subgroups of patients who exhibited under- and overstaging were determined based on the pathological staging. A logistic regression analysis was used to determine correlations between the accuracy rates and patient- or tumor-related factors. A one-way ANOVA was used to determine correlations among inflammatory parameters, pathological lymph nodes, and the depth of invasion.

## 3. Results

### 3.1. Patient Characteristics

A total of 155 colon cancer patients were reviewed for this study. Four patients were excluded due to a lack of data on the MMR status, and twelve patients had preoperative evaluations other than abdominal CT scans. Additionally, eight patients had peritoneal metastases according to the pathology reports. Ultimately, a total of 131 CC patients were analyzed. Twenty-six patients exhibited microsatellite instability. The median age was 68 in the pMMR group and 62 in the dMMR group. The gender distribution was similar across the two groups. The BMI values were also comparable between the pMMR and dMMR patients (Table 1).

### 3.2. Clinical and Pathological Characteristics

Our analysis revealed that dMMR colon cancer was predominantly found in the right hemicolon (76.9%, *p* = 0.023). In terms of the differentiation grade, dMMR tumors were more likely to exhibit moderate (80.8%) or low-grade differentiation (19.2%, *p* = 0.01) and they had higher rates of mucinous differentiation (46.2%, *p* = 0.001). The distribution of pathological T and N staging was similar between the pMMR and dMMR groups (*p* = 0.993, *p* = 0.726) (Table 1).

In our study, most of the tumors were stage T3, with a similar distribution across the pMMR (68.6%) and dMMR (69.2%) groups. Additionally, 61.9% of the pMMR tumors and 53.8% of the dMMR tumors had no lymph node metastasis. N2 lymph node metastasis was present in 14.3% of the pMMR patients and in 19.2% of the dMMR patients (Table 1).

### 3.3. Inflammatory Markers

The median baseline peripheral blood neutrophil and platelet counts at diagnosis were significantly higher in the dMMR group than in the pMMR group (*p* = 0.022 and *p* = 0.022). The median baseline peripheral blood CRP levels were also significantly higher in the dMMR group (*p* = 0.018) (Table 1). The lymph node status did not affect the CRP, neutrophil, or platelet levels in dMMR CC. Although statistically insignificant, a steady increase in the CRP level was observed with increased N status (Table 2). The depth of invasion influenced the CRP levels in dMMR colon cancer (*p* = 0.015). T1 and T2 CC patients had lower CRP levels than T4 CC patients (*p* = 0.025). T3 colon cancer patients had lower CRP levels than T4 CC patients (*p* = 0.028) (Table 2).

### 3.4. Radiological Parameters

Radiological evaluations were compared with pathological evaluations to determine the accuracy of preoperative CT staging. The radiological evaluation for the depth of colonic wall invasion was accurate in 58.1% and 38.5% of the pMMR and dMMR CC cases, respectively (*p* = 0.194). Inaccuracies were mostly due to overstaging in both groups (pMMR 32.4% and dMMR 46.2%) (Table 3).

The lymph node status was correctly predicted in 44.8% of pMMR and 50.0% of dMMR tumors. Incorrect evaluations were mostly due to overstaging in both groups. A total of 54 of 105 (51.4%) pMMR patients and 11 of 26 (42.3%) dMMR patients were overstaged. There was no significant difference in the accuracy in predicting the N status between the two groups (*p* = 0.56) (Table 3).

In the logistic regression analysis, the accuracy of the T and N statuses was not affected by gender, BMI, microsatellite instability, lesion sidedness, or inflammatory parameters (Table 4).

## 4. Discussion

In our study, we observed low levels of accuracy in radiological preoperative staging for both pMMR and dMMR tumors. Right-sided tumors, moderate and low differentiation, and the presence of a mucinous component were more common in the dMMR group. Increased peripheral blood levels of neutrophils, platelets, and CRP were observed in dMMR CC. The accuracy of T and N staging was not influenced by the MMR status or any tumor- or patient-related factors. The T status and CRP levels were correlated with dMMR CC.

In our cohort, 76.9% of all dMMR tumors were located on the right side of the colon. Similarly, in another study, approximately 80–90% of dMMR sporadic cancers occurred in the proximal colon [31]. We observed that dMMR tumors were commonly poorly differentiated and had a higher rate of mucinous differentiation. Microsatellite instability-high (MSI-H) carcinomas tend to be poorly differentiated [26,32]. They frequently exhibit an extracellular mucinous component [14]. Greenson et al. showed a mucinous presence in at least 50% of the tumor area [26]. In our series, the distribution of the T and N staging was similar in pMMR and dMMR CC. However, in other series, a decreased likelihood of metastasizing to regional lymph nodes has been observed in dMMR CC, regardless of the depth of tumor invasion [30,32].

The primary technique for the preoperative staging of colon cancer is an abdominal CT scan [33]. It is easily accessible and comfortable for patients compared to magnetic resonance imaging. However, within the domain of neoadjuvant therapy, its accuracy rates are uncertain [34]. In our group, the accuracy of T and N staging for pMMR and dMMR patients was 58.1% and 38.5% and 44.8% and 50%, respectively. In line with our observations, low confidence levels for T and N staging in both high- and low-stage tumors have been reported in high-volume multicenter cohorts and single-institution studies. In a study on 59,558 CC patients from the Dutch Colorectal Audit database, the accuracy rate for the differentiation of cT3–T4 from cT1–T2 was 75%, and the accuracy in detecting lymph node metastases (N1–2 vs. N0) was 69%. Moreover, the accuracy rates did not significantly improve during the ten-year study period [24]. Both high and low T stages have similarly low accuracy rates. In a study of fifty Spanish centers with 1950 patients, the accuracy of staging was 57% for T4 tumors [35]. In another study, the overall accuracy was 62% for pT3/4 and 68% for early tumors (pT1/2) based on data from the Cleveland Clinic, which included 150 right-sided CC patients. An accuracy rate of 60% for the N status was observed in the same study [36]. Radiological understaging, as well as overstaging, was encountered. A Swedish cohort study investigating 4849 patients with early CC showed that 50% of clinical T1–2 CC cases were actually T3 and 9% were T4. Clinical lymph node overstaging occurred in 52% of cases involving pathological lymph node-negative patients [37].

In our patients, MSI CC did not affect the concordance among CT scans and pathological examinations regarding both T and N staging. The limited number of studies evaluating the effect of microsatellite instability on the accuracy of abdominal CT have yielded consistent results. The MMR status was correlated with the risk of N status overstaging in a retrospective evaluation of 6102 patients in the Danish Colorectal Cancer Group database [28]. Moreover, Platt et al. observed that dMMR tumors assigned an incorrect N status were more likely to be overstaged than pMMR tumors (90% vs. 59%) [29]. In another study, the correlation among the pre- and postoperative N categories was inferior (*p* > 0.05) in dMMR cancers compared to the significant correlation (*p* < 0.01) observed in pMMR cancers [27]. On the other hand, high specificity in predicting pN0 was achieved in MSI-high tumors if a smaller lymph node long axis and a lower CT probability of lymph node metastasis were observed [38]. We observed that overstaging was more common regarding both the T and N statuses, although similar distributions were noted in both pMMR and dMMR patients.

MSI causes a profound inflammatory response in tumors [13], resulting in larger tumor and lymph node sizes [30,38], which may complicate radiological evaluations. Significant histopathological characteristics have been identified in MSI colorectal cancer. Higher rates of lymphocytic infiltration, Crohn’s-like reactions, and peritumoral and intraepithelial lymphocytes have been observed in several studies [12,14,32]. MSI CC commonly exhibits an expanding margin rather than an infiltrative one [39]. Increased long- and short-axis measurements [38], higher cutoff sizes for lymph nodes, and a greater number of lymph nodes with long diameters ≥ 8 mm on abdominal CTs were observed in dMMR patients [11]. Hong et al. observed a smaller proportion of T4 tumors in dMMR CC, and T3 tumors were larger in size in right-sided dMMR CC. A similar distribution of T staging but with an increased tumor size was observed in another study [11,30].

We noted higher neutrophil and platelet counts and CRP levels in dMMR tumors. JR Platt et al. observed increased inflammatory markers in dMMR CC. Moreover, they demonstrated a correlation with a positive N status, as well as the CRP, neutrophil, and platelet counts, in dMMR tumors [29]. In contrast, we observed a correlation with the T staging and CRP levels in dMMR CC. We observed higher CRP levels with an increased depth of colonic wall invasion, likely due to the increased inflammatory response to the tumor associated with MSI CC. Increased CRP levels, and their association with a larger tumor size, lymph node, or liver metastasis; an advanced Dukes’ stage [40]; and decreased survival, have been demonstrated elsewhere [41]. To the best of our knowledge, the relationship among microsatellite status, inflammatory markers, and tumor staging has not been specifically evaluated in other studies.

Our study had a few limitations. Lower T stages were not well represented in this study. Moreover, 68.6% and 69.2% of the pMMR and dMMR patients were pathologically T3. We did not observe increased lymph node status overstaging in dMMR CC in our study cohort. The number of patients may not have been sufficient to demonstrate this divergence. We also retrospectively analyzed a study population of surgically resected CC patients who had undergone contrasted abdominal CT and had a pathological MMR status. Thus, considerable selection bias might have been present.

In conclusion, microsatellite-instable colon cancer has characteristic features, such as right-sidedness, lower differentiation, and mucinous components. We observed increases in inflammatory markers, which were correlated with colonic wall invasion. These findings should be confirmed in further studies. We did not observe differences in the concordance of CT scans and pathological evaluations for both the T and N stages in the pMMR and dMMR groups. Studies consisting of more detailed radiological evaluations of the lymph nodes and colonic wall invasion would illuminate the specific characteristics of dMMR tumors in preoperative settings. From an NAC perspective, the preoperative radiological evaluation of dMMR patients should be conducted separately from those with microsatellite-stable CC. The increased peripheral blood inflammatory parameters may reflect a more advanced disease stage in dMMR CC and could serve as a predictive tool for more invasive carcinomas.

## Figures and Tables

**Table 1:** Patient characteristics, clinical and tumor pathological features, and inflammatory markers.

	pMMR	dMMR	*p*-Value
Age *	68 (58–75)	62 (51.75–72.25)	0.114
**Gender (woman)**	40 (38.1%)	13 (50%)	0.275
BMI, kg/m^2^ *	25 (24–29)	26 (24–31)	0.481
**Side of the tumor**			
**Right**	56 (53.3%)	20 (79.9%)	0.023
**Grade of the tumor**			
**Good**	14 (13.3%)	0	0.01
**Mediate**	86 (81.99%)	21 (80.7%)	
**Poor**	5 (4.8%)	5 (19.3%)	
**Mucinous component**			
**Present**	16 (15.2%)	12 (46.2%)	0.001
**Pathological depth of invasion**			
**T1**	4 (3.8%)	1 (3.8%)	0.993
**T2**	10 (9.5%)	2 (7.7%)	
**T3**	72 (68.6%)	18 (69.2%)	
**T4**	19 (18.1)	5 (19.2%)	
**Pathological lymph node invasion**			
**N0**	65 (61.9%)	14 (53.8%)	0.726
**N1**	25 (23.8%)	7 (26.9%)	
**N2**	15 (14.3%)	5 (19.2%)	
**Inflammatory markers**			
CRP, mg/L *	5.8 (3.45–13.10)	23.98 (3.65–32.0)	0.018
Platelet, 10^3^/mm^3^ *	307 (245–382.5)	387 (261.5–500.0)	0.022
Neutrophil, 10^3^/mm^3^ *	4.6 (3.06–5.70)	5.62 (4.06–6.72)	0.022

* Values are presented as median (IQR), and other values are presented as percentage; *p* = 0.05 is statistically significant. pMMR, microsatellite repair-proficient; dMMR, microsatellite repair-deficient.

**Table 2:** Comparison of inflammatory markers according to T and N staging in dMMR tumors.

	**pN0**	**pN1**	**pN2**	***p*-Value**
**CRP, mg/L**	18.48 ∓ 16.95	24.47 ∓ 21.27	36.68 ∓ 46.64	0.337 ^a^
**Platelet, 10^3^/mm^3^**	387.71 ∓ 118.37	366 ∓ 141.18	374 ∓ 174.64	0.939 ^b^
**Neutrophil, 10^3^/mm^3^**	5.19 ∓ 1.34	6.66 ∓ 2.24	5.38 ∓ 1.44	0.169 ^c^
	pT1,2 *	**pT3**	**pT4**	***p*-Value**
**CRP, mg/L**	4.90 ∓ 5.35	19.62 ∓ 16.81	51.12 ∓ 40.86	0.015 ^d^
**Platelet, 10^3^/mm^3^**	364.0 ∓ 285.67	362 ∓ 129.50	449.20 ∓ 85.26	0.429 ^e^
**Neutrophil, 10^3^/mm^3^**	5.86 ∓ 4.22	5.32 ∓ 1.35	6.84 ∓ 1.80	0.211 ^f^

Values are presented as mean ∓ SD. *P*-value < 0.05 is statistically significant. CRP, C-reactive protein; p, pathological. ^a^ CRP pN0 versus pN1, *p* = 0.870; pN0 versus pN2, *p* = 0.305; pN1 versus pN2, *p* = 0.618. ^b^ Platelet pN0 versus pN1, *p* = 0.938; pN0 versus pN2, *p* = 0.974; pN1 versus pN2, *p* = 0.995. ^c^ Neutrophil pN0 versus pN1, *p* = 0.154; pN0 versus pN2, *p* = 0.974; pN1 versus pN2, *p* = 0.395. * Due to limited number of patients, T1 and T2 tumors were analyzed together. ^d^ CRP pT1,2 versus pT3, *p* = 0.552; pT1,2 versus pT4, *p* = 0.025; pT3 vs. pT4, *p* = 0.028. ^e^ Platelet pT1,2 versus pT3, *p* = 1.00; pT1,2 versus pT4, *p* = 0.635; pT3 vs. pT4, *p* = 0.409. ^f^ Neutrophil pT1,2 versus pT3, *p* = 0.992; pT1,2 versus pT4, *p* = 0.491; pT3 vs. pT4, *p* = 0.187.

**Table 3:** Distribution of radiological accuracy according to pathological stages.

	pMMR	dMMR	*p*-Value
	*n* = 105	*n* = 26	
**Accuracy of T**			
**Correct**	61 (58.1%)	10 (38.5%)	0.194
**Overstaged**	34 (32.4%)	12 (46.2%)	
**Understaged**	10 (9.5%)	4 (15.3%)	
**Accuracy of N**			
**Correct**	47 (44.8%)	13 (50%)	0.56
**Overstaged**	54 (51.4%)	11 (42.3%)	
**Understaged**	4 (3.8%)	2 (7.7%)	

Values are presented as percentages. *p*-value < 0.05 is statistically significant. pMMR, mismatch repair-proficient; dMMR, mismatch repair-deficient.

**Table 4:** Logistic regression analyses for accuracy of T and N stages.

	Odds Ratio	95% CI		*p*-Value
		Lower	Upper	
**Accuracy of T**				
**dMMR**	0.577	0.199	1.669	0.31
**Gender**	1.174	0.512	2.694	0.705
**Age**	0.979	0.948	1.012	0.207
**BMI**	0.97	0.875	1.075	0.563
**Mucinous c**	0.871	0.314	2.416	0.791
**Tumor side**	1.536	0.654	3.608	0.324
**CRP**	0.988	0.962	1.014	0.348
**Platelet**	1.002	0.998	1.006	0.312
**Neutrophil**	1.204	0.876	1.653	0.251
**Accuracy of N**				
**dMMR**	0.154	0.023	1.033	0.054
**Gender**	0.823	0.214	3.168	0.777
**Age**	1.032	0.977	1.091	0.259
**BMI**	1.024	0.865	1.213	0.784
**Mucinous c**	1.5	0.295	7.639	0.625
**Tumor side**	0.744	0.186	2.981	0.676
**CRP**	0.962	0.897	1.032	0.282
**Platelet**	0.996	0.988	1.004	0.359
**Neutrophil**	0.989	0.556	1.701	0.971

*p*-value = 0.05 is statistically significant. BMI, body mass index; CRP, C-reactive protein; mucionuos c, mucinous component; dMMR, mismatch repair-deficient.

## Data Availability

Data are available from the corresponding author upon reasonable request.
